# Lumpy skin disease outbreak in cattle population of Chattogram, Bangladesh

**DOI:** 10.1002/vms3.524

**Published:** 2021-05-16

**Authors:** Farazi Muhammad Yasir Hasib, Mohammad Sirazul Islam, Tridip Das, Eaftekhar Ahmed Rana, Mohammad Helal Uddin, Mohammad Bayzid, Chandan Nath, Mohammad Alamgir Hossain, Mohammad Masuduzzaman, Shubhagata Das, Mohammad Abdul Alim

**Affiliations:** ^1^ Department of Pathology and Parasitology Faculty of Veterinary Medicine Chattogram Veterinary and Animal Sciences University Chattogram Bangladesh; ^2^ Poultry Research and Training Centre Chattogram Veterinary and Animal Sciences University Chattogram Bangladesh; ^3^ Department of Microbiology and Veterinary Public Health Faculty of Veterinary Medicine Chattogram Veterinary and Animal Sciences University Chattogram Bangladesh; ^4^ Department of Medicine and Surgery Faculty of Veterinary Medicine Chattogram Veterinary and Animal Sciences Chattogram Bangladesh; ^5^ School of Animal and Veterinary Sciences Charles Sturt University Wagga Wagga NSW Australia

**Keywords:** cattle, Chattogram of Bangladesh, lumpy skin disease, phylogenetic analyses, prevalence

## Abstract

**Background:**

Lumpy skin disease (LSD) is an important viral disease causing significant economic losses in commercial livestock production. In mid‐2019, an outbreak of LSD has been reported in cattle population from different parts of Bangladesh including Chattogram division. A cross‐sectional surveillance study was undertaken from August 2019 to December 2019 to investigate the prevalence and associated risk factors of LSD in cattle in Chattogram district.

**Methods:**

A total of 3,327 cattle from 19 commercial farms were examined for the LSD specific skin lesions and associated risk factors. A total of 120 skin biopsies were collected from the suspected animal for the confirmation of the disease using molecular detection and histopathological examination. Partial genome sequencing and phylogenetic analyses were performed on selected viral isolates.

**Results:**

The overall clinical prevalence of LSD in the study population was 10% (95% confidence interval [CI]: 9.4%–11%) where the highest farm level outbreak frequency was 63.33% (95% CI: 45.51%–78.13%) and the lowest 4.22% (95% CI: 3.39%–5.25%). Crossbred and female cattle showed a significantly higher prevalence of the disease compared to their counterparts. Introduction of new animals in farms was found to be one of the most significant risk factors in the transmission of the disease. All suspected skin biopsies were positive for LSD virus (LSDV) infection with granulomatous and pyogranulomatous dermatitis was revealed on histopathology. Phylogenetic analysis based on the inverted terminal repeat region of the LSDV gene suggested that the locally circulating strain was closely related to the strains isolated from the Middle East and North African countries.

**Conclusions:**

The data generated in this study would be beneficial to the field veterinarians and animal health decision makers in the country as well as it will aid in taking appropriate measures to prevent further relapse or outbreak of this disease in future.

## Graphical abstract

Lumpy Skin Disease (LSD) is an important viral disease capable of incurring significant economic losses in commercial livestock production. This investigation summarises the clinical outbreaks of LSD in the commercial cattle population in Chattogram district of Bangladesh unrevealing the disease burden and associated risk factors.

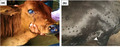

## 1 INTRODUCTION

Lumpy skin disease (LSD) is a viral disease caused by *LSD* *virus* (LSDV) that belongs to the family *Poxviridae* and genus *Capripoxvirus*. The disease affects a wide range of domestic animals including cattle, buffalo, sheep and goats (Alkhamis & VanderWaal, 2016; El‐Nahas et al., 2011), and the main symptoms are fever and nodular lesions on the skin, mucous membrane of respiratory and digestive tracts (Coetzer & Tuppurainen, 2004). The World Organization for Animal Health (OIE) included the disease in notifiable transboundary disease list due to its substantial economic losses in terms of reduced productivity, poor hide quality, poor growth rate, infertility and even death (Anonymous, 2021; Tuppurainen et al., 2017; Tuppurainen & Oura, 2012). LSDV is believed to be transmitted mainly biting arthropods such as mosquitoes, flies and ticks (Magori‐Cohen et al., 2012). Higher incidence of this disease is observed in crossbred young animals with communal grazing and during the wet season when the arthropods vectors are abundant. Introduction of new animals is another important risk factor (Al Rammahi & Jassim, 2015; Alemayehu et al., 2013; Chihota et al., 2001; El‐khabaz, 2014; Kiplagat et al., 2020; Ochwo et al., 2019). Zambia is the first country where LSD was first identified in 1929 that was followed by many African and Middle Eastern countries (Kasem et al., 2018). Although many countries have experienced the outbreak of LSD, but which strain or variants will be the perfect match for vaccine production is remained as a debatable issue (Ayelet et al., 2013; Ben‐Gera et al., 2015). Some recent articles claimed the potentiality of vaccine candidates for LSD prevention (Klement et al., 2020; Wolff et al., 2020; Zhugunissov et al., 2020). In contrast, vaccine strains were also found during the disease outbreak in Russia which raised further doubts on the vaccine candidate and its efficacy (Kononov et al., 2019).

In Bangladesh, an outbreak of an unknown syndrome with nodular skin lesions was reported by local veterinary services authority in mid‐2019 in commercial and backyard cattle population in some locations (Anwara, Karnaphuli and Patiya) of Chattogram district (Anonymous, 2019). Same pattern of clinical onset was reported later in different districts of the country (Giasuddin et al., 2020; Khalil et al., 2021). The outbreak report was preliminary confirmed based on clinical signs and later using the reverse transcription polymerase chain reaction (RT‐PCR) test by the Department of Livestock Services (DLS), Bangladesh and notified the disease as LSD to OIE in August, 2019 (Anonymous, 2019). Therefore, a cross‐sectional surveillance study was undertaken on clinically suspected LSD cases throughout Chattogram district; the south‐eastern part of Bangladesh. The aim of the present study was to confirm the disease occurrence based on clinical, molecular and pathological identification and unveiled the plausible risk factors of LSDV infection in this region. We further analysed the sequence data of the circulating LSDV strains to identify the probable geographical origin of this strain.

## 2 MATERIALS AND METHODS

### 2.1 Study design

The study was conducted over a period of 5 months (August to December 2019) in Chattogram District of Bangladesh at the onset of a lumpy skin disease outbreak. A cross‐sectional study was designed to collect the samples, and individual animal was considered as the sampling unit. A standard questionnaire was used to collect demographic data such as breed, age, sex and other data (e.g., introduction of new animals, source of water supply in the farm, etc.). Selected animals were categorized as Holstein Friesian crossbred (*Bos taurus* X *B. indicus*) and zebu cattle (*B. indicus*). Age of the animals was categorized as calf: ≤1 year; heifer: >1 to ≤2.5 years for crossbred and >1 to ≤3.5 years for indigenous cattle; cow: >2.5 years for crossbred and >3.5 years for indigenous cattle and bull (≥1 year) (Alim et al., 2012). Selection of study areas and animals were based on the suspected cases reported by the local veterinarians and physical visit to the farms. A case was considered positive for LSD when an animal showed two or more of the following signs such as nodular characteristic lesions on the skin, fever, lameness, lymphadenopathy, edema and decreased production (e.g., reduction of milk yield) (Magori‐Cohen et al., 2012). A total of 19 commercial farms from Chattogram district (six farms from Pahartali area, three farms from Sitakunda and two farms from each of Chattogram Port, Double Mooring, Hathazari, Panchlaish and Chadgaon area) were selected (Figure 2). Farms comprising less than 15 cattle were excluded from the present survey (Sikder et al., 2012). Sample from affected animals was collected from the individual farms using a simple randomization technique. A farm was considered positive for ectoparasites (flies, ticks, lice) when at least one animal was infested by one of these parasites.

### 2.2 Sample collection and preservation

**FIGURE 1:**
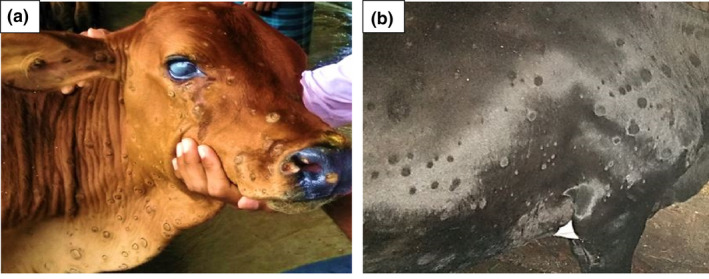
Nodular lesions of LSD affected calf (a) and cow (b)

A total of 19 farms having 3,327 animals were considered consisting of 669 calves, 281 heifers, 2,272 cows and 105 bulls. Data were collected by face to face interview of the animal attendants of the particular farm and physical examination of the cattle. Among the diseased or suspected cattle (Figure 1a,b), a total of 120 skin biopsies from nodular lesions were collected aseptically using punch biopsy techniques (Kasem et al., 2018). Briefly, the biopsy site was shaved by the sterile blades, and a small punch was taken deeply in the skin so that all layers along with the subcutaneous tissue were collected. Half of the skin biopsy specimen was kept in neutral buffered formalin (10%) for histological examination following conventional haematoxylin and eosin (H&E) staining (Fischer et al., 2008). The rest half of the skin biopsy samples were preserved in −20°C for molecular confirmation of the infection.

### 2.3 DNA extraction and PCR presence of LSDV

Total genomic DNA was extracted from all suspected skin biopsies using commercially available kits following manufacturer's instruction with some modifications (DNeasy Blood & Tissue Kits®). PCR was then performed to confirm the presence of LSDV using a set of published primers (forward; GTGGAAGCCAATTAAGTAGA and reverse; GTAAGAGGGACATTAGTTCT) targeting the inverted repeat region (ITR) of the genome (Stram et al., 2008). In brief, PCR reactions were set up in 50‐μl final volumes containing 25‐μl master mix, 2.5‐μl forward primer, 2.5‐μl reverse primer, 5.0‐μl DNA template and 15‐μl nuclease free water. The PCR conditions were follows as an initial denaturation step of 95°C for 1 min followed by 35 cycles of denaturation at 94°C for 30 s, annealing at 49°C for 30 s, extension at 72°C for 70 s and a final extension step at 72°C for 5 min. Then, 5‐μl of amplified amplicons were taken and stained using 0.05% ethidium bromide (Sigma‐Aldrich^®^) and a visualization of the band (1,237 bp) after agarose gel (1%) electrophoresis.

### 2.4 Nucleotide sequencing and phylogenetic analysis

Four randomly selected LSDV PCR amplicons were gel purified using Wizard® SV Gel and PCR Clean‐Up System (Promega) and sequenced by sanger dideoxy sequencing (Macrogen^®^). The sequence read data were then manually cleaned up using the chromatogram software (Geneious Prime version 2020) and deposited in GenBank. NCBI BLAST was performed for each of the sequence reads and the ITR from a diverse range related LSDV and other poxvirus genome sequences (Nt sequence identity 100%–70%) were retrieved (*N* = 63) and aligned with MAFFT v7.017 using G‐INS‐i (gap open penalty 1.53; offset value 0.123) alignment algorithm (Katoh et al., 2002). jModelTest program 2.1.3 favoured a general‐time‐reversible model with gamma distribution rate variation and a proportion of invariable sites (GTR + I + G4) for the phylogeny (Darriba et al., 2012). Maximum likelihood (ML) phylogenetic trees was reconstructed using the program PhyML v3.1 (Guindon & Gascuel, 2003), and FigTree v1.4 was used to generate the consensus tree (Smith et al., 2009). The proportion of bootstrap support (%) was showed in each branch while multiple taxa showing polytomy, and closely related isolates were collapsed for better visualization.

### 2.5 Statistical analysis

All data were inserted and coded in Microsoft office Excel 2016 spreadsheet, and both univariable and multivariable analyses were performed using generalized linear mixed models in STATA‐IC 13. Farm was included in the model as random effect. Backward elimination procedure was followed, and a *p* value ≤ 0.05 was considered significant in both univariable and multivariable analyses. Prevalence map along with location and size of the farms was created using QGIS 3.12.0.

## 3 RESULTS

### 3.1 Clinical prevalence of LSD

**FIGURE 2:**
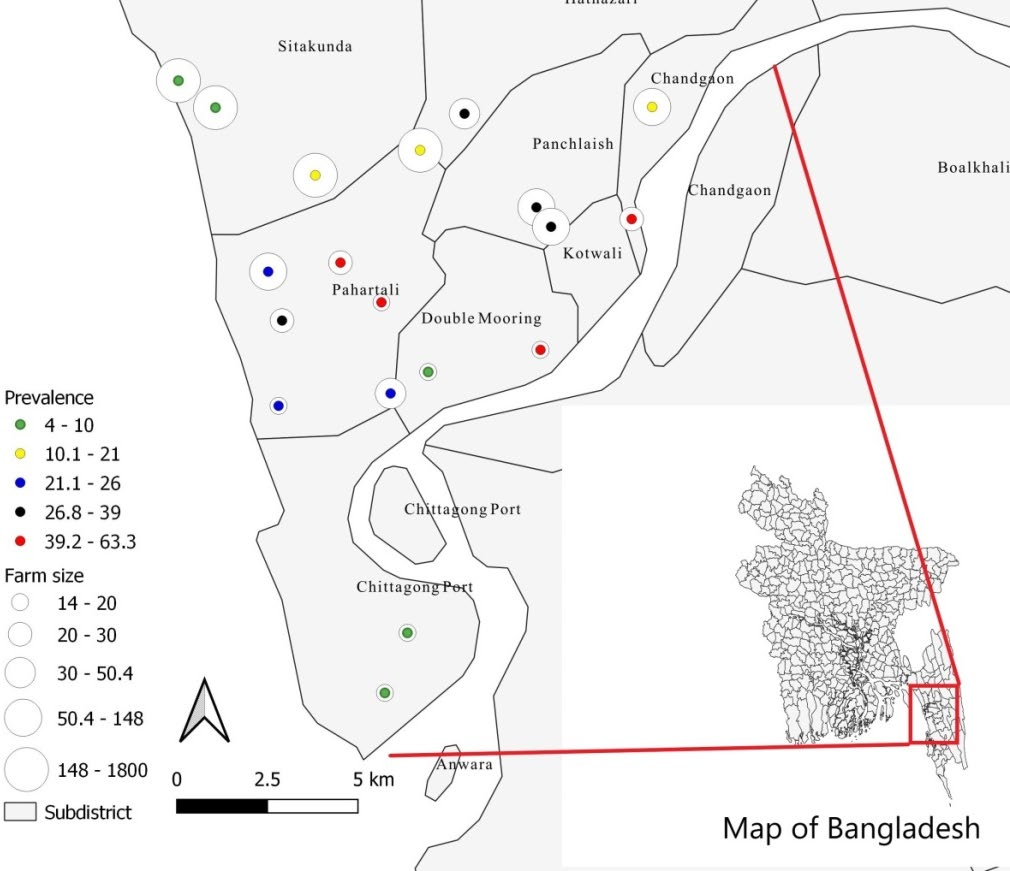
Map showing the location and farm size (circle) along with the number infected animals (farm level frequency, %) in each farm

The overall clinical prevalence of LSD was 10% (95% CI: 9.4%–11%) in the study farms. The farm level highest frequency was 63.33% in one of the farms located in Chadgaon region, and the lowest was 4.22% in a farm at Sitakunda region of Chattogram District (Figure 2). The clinical prevalence ranges from 20% to 40% in five farms of Pathartali, two farms of Panchlaish, and one farm of Hathazari and Chandgaon area of Chattogram district. This clinical prevalence ranges 41%–63% in two farms of Chattogram port area and one farm of Pahartali, Double mooring and Chandgaon area. Three farms of Sitakunda and one farm of Pahartali and Double mooring area of the same district showed the clinical prevalence below 10% (Figure 2).

### 3.2 Risk factors associated with the occurrence of LSD

The clinical prevalence was observed the lowest in bulls (5%) (95% CI: 2%–10%). Univariable analysis showed that odds ratio (OR) of having the disease in calves, cows and heifers were 1.37 (CI: 0.53–3.55), 2.52 (CI: 1.02–6.26) and 3.51(CI: 1.35–9.14) times higher compared to bulls, respectively (Table 1). Females were in higher risk (OR = 2.26, CI: 1.28–4.0) than males. In terms of lactation, with increasing lactation number decrease in prevalence was observed; odds of having the disease in first lactation was 7 times higher compared to fourth lactation. The univariable analysis also showed that local cattle were less susceptible than the crossbred. Besides, introduction of new animals, sources of water supply and floor types (brick or cemented) in the farm act as potential risk factors of the disease (Table 1). In multivariable model, crossbred (*p* = .0080, OR = 3.58, CI: 1.40–9.17) and female (*p* ≤ .0001, OR = 3.96, CI: 2.16–7.27) cattle had a significantly higher risk of getting the disease compared to their counterparts (Table 2).

**TABLE 1:** Risk factors associated with lumpy skin disease in cattle farms of Chattogram district of Bangladesh from the univariable logistic regression analysis

Variables	Level	*N* (animals)	Positive *N (%)*	OR (95% CI)	*p* value
Breed	Cross	3,220	340 (11)	2.40 (0.97–5.95)	.05
	Local	107	5 (5)	Ref	
Animal category	Calf	669	43 (6)	1.37 (0.53–3.55)	<.0001
	Heifer	281	42 (15)	3.51 (1.35–9.14)	
	Cow	2,272	255 (11)	2.52 (1.02–6.26)	
	Bull	105	5 (5)	Ref	
Sex	Female	3,071	332 (11)	2.26 (1.28–4.00)	0
	Male	256	13 (5)	Ref	
Lactation^a^	1	107	98 (92)	7.70 (6.04–9.37)	<.0001
	2	267	105 (39)	4.25 (2.77–5.73)	
	3	1,780	50 (3)	1.10 (0.37–2.57)	
	4	118	2 (2)	Ref	
Introduction of new animals	Yes	62	13 (21)	2.34 (1.25–4.36)	.007
	No	3,265	332 (10)	Ref	
Water source	Pond	50	14 (28)	3.46 (1.84–6.48)	<.0001
	Underground (tubewell)	3,277	331 (10)	Ref	
Floor	Brick	72	13 (18)	1.93 (1.05–3.57)	.03
	Cemented	3,255	332 (10)	Ref	
Overall		3,327	345 (10)		

Note

Abbreviations: CI, confidence interval; OR, odds ratio.

^a^ This OR was calculated only including lactating cows.

**TABLE 2:** Risk factors associated with lumpy skin disease in cattle farms of Chattogram district of Bangladesh using a logistic regression analysis

Variables	Level	Estimates	*SEM*	OR (95% CI)	*p* value
Intercept		−3.71			
Breed	Cross	1.277	0.605	3.58 (1.40–9.17)	.0080
	Local	0		Ref	
Sex	Female	1.377	0.479	3.96 (2.16–7.27)	<.0001
	Male	0		Ref	
Random effect of farm		1.003	0.186		

Abbreviations: CI, confidence interval; OR, odds ratio; *SEM*, standard error of the mean.

### 3.3 Molecular identification of LSDV

**FIGURE 3:**
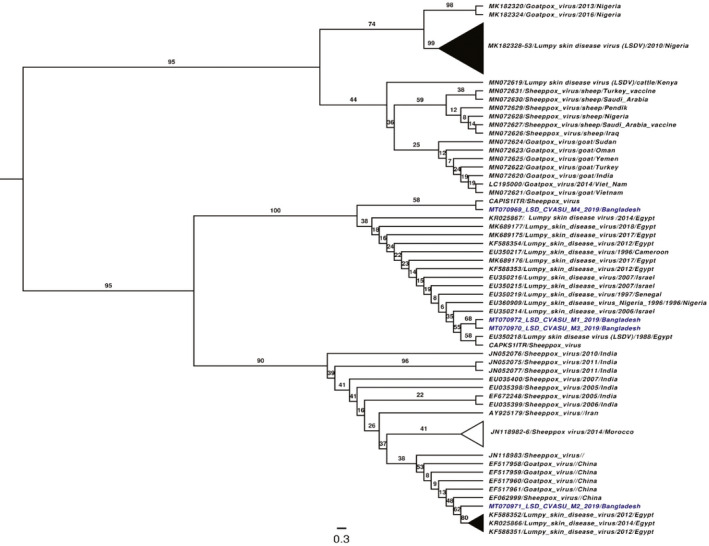
Maximum likelihood (ML) tree rooted at midpoint with proportionally arranged branches based on the ITR region of poxvirus genome demonstrating phylogenetic relatedness of LSDV isolates form Chattogram, Bangladesh (blue taxa). Clades suggesting polytomies were collapsed, and shown in cartoon, the bootstrap statistics (percentage) were shown as branch support numbers

All of the collected skin biopsies were PCR positive for LSDV. Among them, a total of 4 samples were sequenced randomly (GenBank accession: MT070969‐72). The ML tree reconstructed from the ITR region of closely related poxviruses revealed that most LSD_CVASU isolates belong to a strongly supported (100% bootstrap value) clade dominated by LSDV strains. LSDV isolated from different regions of the world (Africa and Middle East) over three decades (1997–2019) timeframe. Isolate LSD_CVASU_M1 and M3 (MT070969 and MT070971) together formed a sister branch to the LSDV isolate from Egypt (EU350218) with a moderate bootstrap support (55%), while for isolate M4 (MT070972), the phylogenetic resolution was not clear and demonstrated some relatedness (58% bootstrap support) with sheep pox reference sequence (CAPIS1ITR) (Figure 3). On the other hand, Isolate M2 on the other hand showed stronger bootstrap support (80%) towards recent isolates of LSDV from Egypt (KF588352, KR052866 and KF58835). The phylogenetic reconstruction thus reaffirm that the viral isolates from the nodular skin biopsies were LSDV genotypes most closely related to those from Egypt (Figure 3).

### 3.4 Histological features of the skin biopsies

**FIGURE 4:**
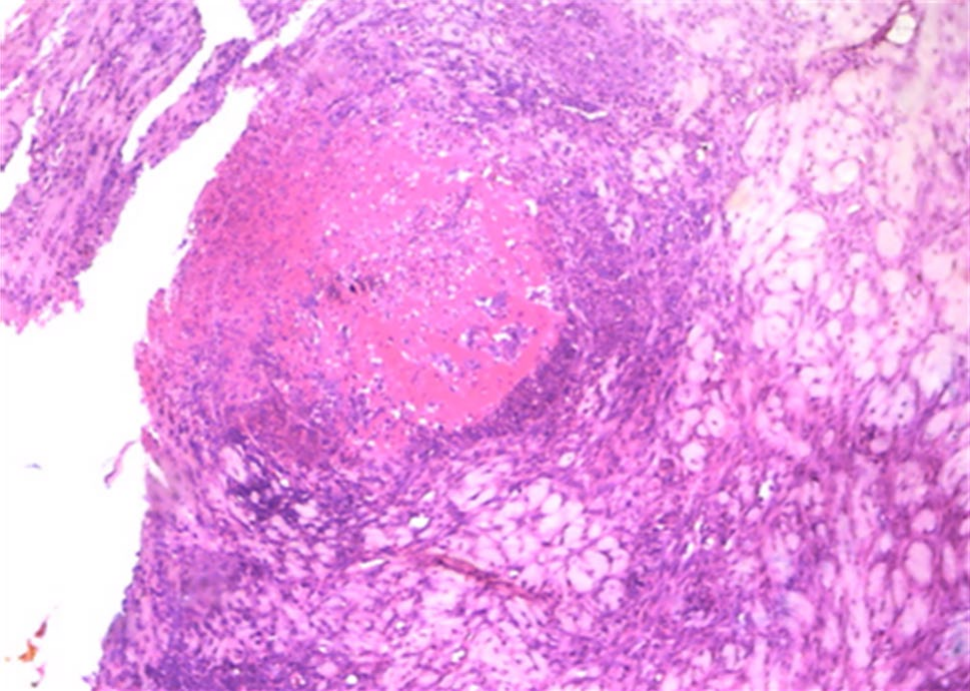
Histological feature of LSD affected nodular lesions. Figure demonstrated deep dermis and subcutis; focal granulomatous lesion comprised of necrotic debris and encircling mononuclear cell infiltration (magnification of image 100×)

All cases involved granulomatous and pyogranulomatous dermatitis with multifocal to diffuse deep dermal necrosis and panniculitis (Figure 4). The superficial and deep dermis was infiltrated with variable numbers of lymphocytes, plasma cells, macrophages and relatively fewer neutrophils. However, multifocal small dermal abscesses also observed in the samples. Acanthosis and orthokertatotic hyperkeratosis were common features of the nodules with instances of epidermal ulceration. Hair follicles of the skin biopsies were partially destroyed and replaced by necrotic epithelium, mixed cellular infiltrates and keratinaceous debris from ruptured follicles (furunculosis) (Figure 4). Pannicular infarction and subcutaneous vasculitis were present in the samples.

## 4 DISCUSSION

Bangladesh was free of LSD before mid of 2019, and the very first LSDV infection was reported in Anwara, Karnaphuli and Patiya Upazila (subdistrict), Chattogram to OIE in August 2019 (Anonymous, 2019). The present investigation summarises the clinical outbreaks of LSD in the commercial cattle population in Chattogram District unrevealing the disease burden and associated risk factors. The epidemiological data were supported by histopathological features of the clinically characteristic nodular skin lesions as well as PCR‐based molecular identification and phylogenetic analyses.

The overall clinical prevalence of LSD in Chattogram District was 10% similar to some previous studied in Saudi Arabia, Ethiopia and Turkey who reported 6%–12% prevalence in their cattle population (Abera et al., 2015; Al‐Salihi & Hassan, 2015; Kasem et al., 2018; Şevik & Doğan, 2017). Body et al., (2012) observed a much higher prevalence (27.9%) in cattle of Oman which was higher than the overall prevalence of the current study. The higher or lower prevalence of disease might have been influenced by many factors such as geography, farm management and biosecurity, seasons, availability of arthropods vectors, importation of animal from infected areas, disposal of the dead animals. Although we didn't observe any mortality in the study population, some of the previous studies reported 0.99%–2.12% of mortality (Gari et al., 2010; Kasem et al., 2018). Comparatively shorter duration of the actual study period and culling of diseased animals might be a reason for the paucity of mortality. However, clinical form of LSD is generally associated with economic loss in terms of production and treatment expenditure (Babiuk et al., 2008a; 2008b).

Risk factors analyses suggests that crossbred cattle were more susceptible to LSD than indigenous cattle which was consistent with the findings of previous studies (Al Rammahi & Jassim, 2015; Kiplagat et al., 2020; Klement et al., 2018). Higher susceptibility of crossbred cattle might be due to lower disease resistance capability in comparison to indigenous breeds (Tageldin et al., 2014). Further, the higher number of crossbred animals (96.79%) was sampled over local (3.21%) cattle might explain the variation of the results. Heifers were affected largely with LSD in comparison to bulls, calves and cows. In previous studies, a higher morbidity was recorded in younger cattle (<2 years) in Saudi Arabia (Kasem et al., 2018) and calves (0.5–1 year) in Ethiopia (Molla et al., 2018). This might be due to management system of the farms where heifer was kept in poor hygienic conditions in comparison to other animals (calf, cow or bull). Females were more prone to LSD compared to males which was consistent with previous research (Ayelet et al., 2014; Magori‐Cohen et al., 2012; Salib & Osman, 2011). Higher frequency of LSD in female cattle could be due to their exposure to many stress conditions, e.g., pregnancy, parturition and sometimes less amount of feed supplied compared to their actual requipment (Kasem et al., 2018). We observed an inverse relationship with lactation number in the occurrence of LSD in cattle although we were unable to identify the possible reasons. Farm‐specific risk factors such as introduction of new animals to the farm demonstrated a significantly higher risk to be infected with the virus or its transmission which was supported by previous findings (Gari et al., 2010; Macpherson, 1994; Munyeme et al., 2008).

The histopathological features of the suspected nodular skin biopsies demonstrated granulomatous and pyogranulomatous dermatitis with vasculitis and pannicular involvement (Figure 4) which are merely non‐specific lesions. However, similar histological features of suspected skin lesions were documented in many prior studies with confirmed LSD cases (Abdallah et al., 2018; Body et al., 2012; El‐khabaz, 2014; Stram et al., 2008). Unsurprisingly, we could not identify any intracytoplasmic inclusion bodies or so‐called ‘sheep pox’ cells (SPCs) or ‘cellules claveleuses’ of Borrel in any of the skin biopsies (El‐Neweshy et al., 2013). Although SPCs or intracytoplasmic inclusions often considered confirmatory histopathological findings for LSD (Abdallah et al., 2018; Body et al., 2012), they are rarely found in natural LSD cases (El‐Neweshy et al., 2013; House et al., 1990) and often associated only with acute phase infections. In the present study, all tissue sections were stained with H&E, and the histological features leaned towards subacute to chronic stage infections. Future studies should incorporate immunohistochemistry of the tissue section using anti‐LSDV monoclonal antibodies to reveal replicating virus particles in macrophages and epithelial cells of dermis.

The PCR‐based molecular test targeting ITR region of the LSDV has successfully confirmed all suspected cases of LSD in this study, and the local genotype circulating in Chattogram district was deposited in GenBank as well (Gene bank accession no. MT070969‐MT070972). The ML tree reconstructed from the ITR region of all related poxviruses showed that the LSDV strains circulating in Bangladesh are closely related to that in Middle East and North Africa as three out of four sequences had closest phylogenetic relationship with isolates from Egypt. However, the ITR region of the genome used for amplification and sequencing of LSDV is a pseudogene, relatively conserved and homologous to many other poxvirus genomes (Gershoni & Black, 1989). Therefore, the phylogenetic reconstruction had lack of discriminatory resolution, as presented by relatively low bootstrap supports in many branches and positioning LSDV genotypes with sheep pox and goat poxvirus strains in some clades (Figure 3). This limitation might have imposed a negative implication defining the possible source of the outbreak based on evolutionary relatedness of geographically distant strains. Further studies should incorporate sequencing at least three different core gene groups along with concatenation or partitioning approach for alignment and subsequent phylogenetic analyses to reconstruct a comprehensive evolutionary tree with better discriminatory power and resolution.

In Bangladesh, there is no previous outbreak of LSD in any of the susceptible species including cattle. Many factors might have been involved with the current outbreak and transmission across the country. There are both legal and illegal cattle trading occurs every year from neighbouring countries, namely, India and Myanmar. Further, throughout the year, livestock mobility across the country is quite high which usually reaches a peak during Eid‐ul‐Adha (a holy festival of Muslim) as thousands of temporary wet markets are established to meet the demand (Khatun et al., 2016). It is also mentionable that this outbreak was reported just a month after the festival. It is plausible that unregulated and illegal import of live animals without prior health check or quarantine measures have embarked the clinical outbreak of LSD. Unrestricted in‐country movements of livestock even after the first reporting might have significantly aggravated the viral transmission (Tuppurainen et al., 2017). However, outbreak of this disease occurred in China and Odisha of India in August 2019 (Anonymous, 2019; Sudhakar et al., 2020), and this could be an unexplored link to this outbreak as cattle movements were speculated as a risk factor (Klausner et al., 2017). Within farm LSDV transmission is further related with the biosecurity measures and other management practices. We found a positive correlation between the communal water supply as well as the floor made of brick as observed by others (Babiuk et al., 2008a; 2008b; Tuppurainen & Oura, 2012). We have observed ectoparasites in almost all the farms which may play a role in the transmission of this virus as reported by previous research (Ince et al., 2016). Future research should be directed for identification of the specific vectors to overcome the limitation of this study. However, the present study may have some inherent limitations of cross‐sectional study despite designed carefully. Particularly, this study design might lead to selection bias, recall bias and temporal sequences between exposure and outcome cannot be evaluated. We recorded most of the clinical data by visual observation that might have minimized the recall bias and major number of variables would not be affected by the temporal sequence, such as sex, breed, type of animal, etc.

In summary, the current study investigated the outbreak of LSDV infection in commercial farms of Bangladesh unveiling the overall clinical prevalence and risk factors associated the disease. This study also suggests a plausible source of the outbreak based on limited genomic data and evolutionary assays. As there is no effective vaccine of this economically important disease, further research should be focused on the molecular characterization of the whole genome of the local strain of LSDV for developing a suitable vaccine candidate. The data generated in this study would be beneficial to the field veterinarians and animal health decision makers in Bangladesh, and also it will aid in taking appropriate measures to prevent further relapse or outbreak of this disease in future.

## 5 ETHICAL APPROVAL STATEMENT

Ethical approval was obtained from the institutional ethical approval committee of Chattogram Veterinary and Animal Sciences University (CVASU) [CVASU/Dir (R&E) EC/2019/126(13)].

## CONFLICT OF INTEREST

There are no conflicts of interest between the authors.

## AUTHOR CONTRIBUTION

**Farazi Muhammad Yasir Hasib:** Data curation; Investigation; Methodology; Project administration; Visualization; Writing‐original draft; Writing‐review & editing. **Mohammad** **Sirazul Islam:** Data curation; Investigation; Methodology; Project administration; Writing‐original draft. **Tridip Das:** Data curation; Formal analysis; Methodology. **Eaftekhar Ahmed Rana:** Formal analysis; Investigation; Methodology. **Mohammad** **Helal Uddin:** Data curation; Investigation; Methodology. **Mohammad** **Bayzid:** Data curation; Investigation; Methodology. **Chandan Nath:** Data curation; Investigation; Methodology. **Mohammad** **Alamgir Hossain:** Conceptualization; Funding acquisition; Resources; Validation; Visualization; Writing‐review & editing. **Mohammad** **Masuduzzaman:** Conceptualization; Funding acquisition; Resources; Visualization; Writing‐review & editing. **Shubhagata Das:** Conceptualization; Formal analysis; Methodology; Resources; Software; Validation; Writing‐original draft; Writing‐review & editing. **Md Abdul Alim:** Conceptualization; Data curation; Funding acquisition; Investigation; Methodology; Project administration; Supervision; Validation; Writing‐original draft; Writing‐review & editing.

### PEER REVIEW

The peer review history for this article is available at https://publons.com/publon/10.1002/vms3.524.

## Data Availability

Data files associating this research are available online (https://doi.org/10.6084/m9.figshare.12619868.v1).
